# Complementary PTM Profiling of Drug Response in Human Gastric Carcinoma by Immunoaffinity and IMAC Methods with Total Proteome Analysis

**DOI:** 10.3390/proteomes3030160

**Published:** 2015-08-07

**Authors:** Matthew P. Stokes, Charles L. Farnsworth, Hongbo Gu, Xiaoying Jia, Camilla R. Worsfold, Vicky Yang, Jian Min Ren, Kimberly A. Lee, Jeffrey C. Silva

**Affiliations:** 1 Cell Signaling Technology, 3 Trask Lane, Danvers, MA 01923, USA; E-Mails: cfarnsworth@cellsignal.com (C.L.F.); hongbo.gu@cellsignal.com (H.G.); xjia@cellsignal.com (X.J.); vyang@cellsignal.com (V.Y.); jmren@cellsignal.com (J.M.R.); klee@cellsignal.com (K.A.L.); jsilva@cellsignal.com (J.C.S.); 2 Department of Biology, Emory University, Atlanta, GA 30322, USA; E-Mail: camilla.worsfold@emory.edu

**Keywords:** antibodies, LC-MS/MS, phosphorylation, gastric carcinoma

## Abstract

Gaining insight into normal cellular signaling and disease biology is a critical goal of proteomic analyses. The ability to perform these studies successfully to extract the maximum value and discovery of biologically relevant candidate biomarkers is therefore of primary importance. Many successful studies in the past have focused on total proteome analysis (changes at the protein level) combined with phosphorylation analysis by metal affinity enrichment (changes at the PTM level). Here, we use the gastric carcinoma cell line MKN-45 treated with the c-Met inhibitor SU11274 and PKC inhibitor staurosporine to investigate the most efficient and most comprehensive strategies for both total protein and PTM analysis. Under the conditions used, total protein analysis yielded few changes in response to either compound, while analysis of phosphorylation identified thousands of sites that changed differentially between the two treatments. Both metal affinity and antibody-based enrichments were used to assess phosphopeptide changes, and the data generated by the two methods was largely complementary (non-overlapping). Label-free quantitation of peptide peak abundances was used to accurately determine fold-changes between control and treated samples. Protein interaction network analysis allowed the data to be placed in a biologically relevant context, and follow-up validation of selected findings confirmed the accuracy of the proteomic data. Together, this study provides a framework for start-to-finish proteomic analysis of any experimental system under investigation to maximize the value of the proteomic study and yield the best chance for uncovering actionable target candidates.

## 1. Introduction

Proteomic methods are constantly being improved to better understand the signaling events that underlie both normal cellular processes as well as complex disease states such as cancer. In the past, investigation of these signaling pathways was limited to detailed study of only one or a few proteins at a time. Developments in proteomic technologies have facilitated monitoring of entire signaling networks in a single study. It is now possible to study the differences in both total protein levels as well as levels of post-translational modifications (PTMs) of thousands of proteins [1,2,3,4,5,6,7,8,9,10,11,12,13,14,15,16,17,18,19,20,21,22,23,24,25,26,27,28,29,30,31,32,33,34,35,36]. Methods have been successfully developed to study protein phosphorylation, ubiquitination, acetylation, methylation, succinylation, and other lysine-acyl modifications [21,37,38,39,40,41,42,43,44,45,46,47,48] to extend the scope of a particular study depending on the overall goals and objectives of the project. 

Whereas the best enrichment strategies for many PTMs rely on the use of immunoaffinity reagents, for phosphorylated peptides both antibody-based as well as metal affinity enrichment-based methods exist. Phosphorylated peptides can be selectively enriched through interaction of the negatively charged phosphate groups with positively charged metal ions including iron (IMAC), titanium dioxide, and zirconium dioxide [5,6,11,18,27,31,34,36]. Many researchers now routinely employ fractionation of digested cellular proteins or peptides and parallel analysis of total protein levels as well as phosphopeptide levels via metal affinity enrichment [5,8,13,15,17,20,23,24,25,30,31,33,34,35]. These studies have been performed in large-scale and have resulted in identification of thousands of proteins and phosphorylation sites across a wide range of cellular and disease systems. 

Antibodies can be produced to recognize a characteristic sequence motif from a broad range of peptides rather than a single amino acid sequence [49]. These “motif antibodies” can be generated to recognize a particular PTM in a context independent manner, or a more restrictive motif such as a phosphorylated residue within a consensus kinase substrate sequence. These motif antibodies have been incorporated into proteomic methods that have also proven useful in the study of disease signaling. Antibody-based methods have been developed including enrichment of tyrosine-phosphorylated peptides [39,43,44,50,51,52], peptides that share a common consensus kinase substrate motif [41,45,53,54], and peptides that are modified by PTMs other than phosphorylation, for which no metal affinity enrichment exists [37,38,40,42,46]. More recently, immunoaffinity reagents have been generated that target key regulators of known signaling pathways, or critical protein classes such as kinases, allowing multiplexed detection and quantitation of hundreds to thousands of regulatory sites in a single experiment [55,56].

With both metal affinity and antibody-based methods available, it raises the question as to whether either method is sufficient for comprehensive coverage of the phosphoproteome. Previous work has suggested that the two methods provide complementary coverage of phosphorylation sites [53,57,58]. This would be expected, as metal affinity methods will preferentially identify phosphopeptides present at higher levels (abundance-driven), while antibody-based methods will identify peptides that share the sequence characteristics targeted by the antibody itself (affinity-driven). 

To investigate the overlap between antibody-based and metal affinity peptide capture methods, we have characterized the response of the human gastric carcinoma cell line MKN-45 to the c-Met inhibitor SU11274 as well as the more general PKC inhibitor staurosporine [59,60,61,62]. MKN-45 cells are known to depend on c-Met signaling pathways for growth and survival (Met-driven), and PKC signaling pathways are known to be dysregulated in gastric carcinomas [39,63,64]. Here we show that combination of individual motif antibodies into mixed reagents with even broader specificity allows identification of thousands of phosphorylated peptides in a single LCMS run, and that these antibody mixtures provide data that is complementary to IMAC enrichment. The overlap of identified peptides between antibody-based and metal affinity-based strategies was low in all cases, emphasizing the importance of both methods to obtain the most complete phosphoproteome coverage possible. Tyrosine phosphorylation was much more efficiently monitored using the antibody-based method, with thousands of peptides identified compared to only ~200 with metal affinity enrichment. 

Analysis of total protein levels across the samples showed that there were almost no changes in protein level with either treatment, while thousands of changes were observed for phosphopeptides. This emphasizes the importance of profiling beyond total protein levels, depending on the experimental system being investigated. Finally, protein interaction network analysis was performed on the combined data, demonstrating the broad coverage of proteins identified across diverse cellular signaling spaces as well as deep coverage within particular signaling pathways. Together, this study provides a framework for start-to-finish profiling of cellular pathways affected by normal growth and development, disease biology, or inhibitor studies, to extract the most information from biological samples as possible.

## 2. Experimental Section

### 2.1. Overview 

Antibody immunoaffinity purification was performed using the PTMScan method (Figure S1) developed at Cell Signaling Technology, as previously described [43,44,45].

### 2.2. Cell Lines and Tissues

MKN-45 cells were from DSMZ (German Collection of Microorganisms and Cell Cultures). Cells were cultured in RPMI supplemented with 10% fetal bovine serum (FBS) and penicillin/streptomycin at 37 °C with 5% CO_2_. Cells were incubated in 0.2% FBS media for 12 h and treated with 1 µM SU11274 (Sigma, St. Louis, MO, USA, #S9820) or 200 nM Staurosporine (Cell Signaling Technology, Inc., Danvers, MA, USA, #9953) for 2 h. The same volume of DMSO was used as a control. Ten milligrams of protein from each cell line or tissue was used for a single immunoprecipitation. Ten milligrams corresponds to approximately 1 × 10^8^ cells, though absolute amounts are cell line and tissue specific. Six hundred nanograms of total protein for each sample was used for total proteome analysis. 

### 2.3. Cell Lysate Preparation

Cells were washed twice with cold PBS. PBS was removed and cells were scraped in Urea Lysis Buffer (9 M sequanal grade Urea, 20 mM HEPES pH 8.0, 1 mM β-glycerophosphate, 1 mM sodium vanadate, 2.5 mM sodium pyrophosphate). Cells were sonicated 3 times for 20 s each at 15 W output power with a 1-minute cooling on ice between each burst. Sonicated lysates were centrifuged 15 min at 4 °C at 20,000× *g*. An aliquot of each supernatant was reserved for Western blotting and stored at −80 °C. Supernatants were collected and reduced with 4.5 mM DTT for 30 min at 55 °C. Reduced lysates were alkylated with 10mM iodoacetamide for 15 min at room temperature in the dark. Samples were diluted 1:4 with 20 mM HEPES pH 8.0 and digested overnight with 10 ug/mL trypsin-TPCK (Worthington, Lakewood, NJ, USA, #LS003740) in 1 mM HCl or 80 µg LysC (Wako, Richmond, VA, USA, #129-02541) per 5 mg cellular protein. Total protein amounts for each sample were normalized prior to sample digestion to ensure equal protein input for all samples. Digested peptide lysates were acidified with 1% TFA and peptides were desalted over 360 mg SEP PAK Classic C_18_ columns (Waters, Richmond, VA, USA, #WAT051910). Peptides were eluted with 40% acetonitrile in 0.1% TFA, dried under vacuum, and stored at −80 °C.

### 2.4. Immunoprecipitation 

**Figure 1:**
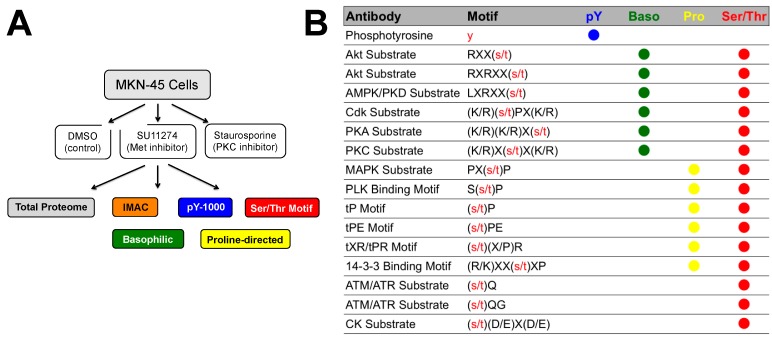
Experimental design and motif antibodies. (**A**) Experimental design: MKN-45 cells were treated with DMSO, the c-Met inhibitor SU11274, or the PKC inhibitor staurosporine. Sufficient quantities of sample were prepared to perform total protein analysis as well as metal affinity enrichment (IMAC) and a variety of antibody-based enrichments. (**B**) Antibodies used in the study along with the recognition motif of each antibody and the mixed reagents in which they were included.

PTMScan motif antibodies were combined as indicated in Figure 1B. Saturating amounts of the indicated antibodies were bound to 40 μL packed Protein A Agarose beads (Roche, Basel, Switzerland) overnight at 4 °C. Lyophilized peptides were resuspended in MOPS IAP buffer (50 mM MOPS pH 7.2, 10 mM KH_2_PO_4_, 50 mM NaCl) and centrifuged 5 min at 12,000 RPM in a MiniSpin microcentrifuge (Eppendorf, Hauppauge, NY, USA). Supernatants were mixed with PTMScan Reagent-Bead slurries 2 h at 4 °C. Beads were pelleted by centrifugation 30 s at 5400 RPM in a MiniSpin microcentrifuge at 4 °C. Beads were washed twice with 1 mL MOPS IAP buffer and four times with 1 mL water (Burdick, and Jackson, Honeywell, Morristown, NJ, USA #AH-365-4). Peptides were eluted from beads with 0.15% TFA (sequential elutions of 65 µL followed by 55 µL, 10 min each at room temperature). Eluted peptides were desalted over tips packed with Empore C_18_ (Sigma) and eluted with 40% acetonitrile in 0.1% TFA. Eluted peptides were dried under vacuum. LysC digested peptides were subjected to a second, in-solution trypsin digest using 250 ng of sequencing grade trypsin (Promega, Madison, WI, USA) in 50 mM ammonium bicarbonate/5% acetonitrile for 2 h at 37 °C. Samples were acidified with TFA and re-purified over C_18_ tips as before.

### 2.5. IMAC

IMAC enrichment was performed as previously described [65]. Nickel-agarose beads (Invitrogen, Carlsbad, CA, USA) were treated with EDTA to remove the Nickel, washed 3 times with H_2_O, loaded with aqueous FeCl_3_ for 30 min, and washed. For phosphopeptide enrichment 10 µL Fe^3+^-agarose slurry was added to 0.5 mg peptide in 1 mL 0.1% TFA/80% acetonitrile for 30 min at room temperature. Unbound peptides were removed by washing 3 times with 0.1% TFA/80% MeCN. Bound peptides were eluted twice sequentially with 50 µL of 2.5% ammonia/50% acetonitrile solution for 5 min and dried in a speed-vac. Samples were resuspended in 100 µL 0.15% TFA + 2 µL 20% TFA, desalted over C_18_ and dried as previously described.

### 2.6. LC-MS/MS Analysis

Immunoprecipitated peptides were resuspended in 0.125% formic acid and separated on a reversed-phase C_18_ column (75 µm ID × 10 cm) packed into a PicoTip emitter (~8 µm ID, New Objective, Woburn, MA, USA) with Magic C_18_ AQ (100 Å × 5 µm). Each sample was split and analytical replicate injections were run to increase the number of identifications and provide metrics for analytical reproducibility of the method. For antibody enrichment peptides from 5 mg protein were run per injection, for IMAC peptides from 167 µg protein were run per injection, and for total proteome peptides from 300 ng were run per injection. Replicate injections were run non-sequentially to reduce artifactual changes in peptide abundance due to changes in instrument performance over time. One replicate of each sample was injected, then the second replicate in reverse order. Peptides were eluted using a 90-min or 150-min linear gradient of acetonitrile in 0.125% formic acid delivered at 280 nL/min. Tandem mass spectra were collected in a data-dependent manner with an LTQ-Orbitrap ELITE mass spectrometer running XCalibur 2.0.7 SP1 (Thermo Scientific, Waltham, MA, USA) using a top-twenty MS/MS method, a dynamic repeat count of one, and a repeat duration of 30 s. Real time recalibration of mass error was performed using lock mass [66] with a singly charged polysiloxane ion *m*/*z* = 371.101237. The data associated with this manuscript may be downloaded from CHORUS using the numbers listed in Supplemental Table S1. The RAW data files are accessible as public data with CHORUS ID numbers as outlined in Table S1. 

MS/MS spectra were evaluated using SEQUEST and the Core platform from Harvard University [15,30,67]. Files were searched against the NCBI *Homo sapiens* FASTA database updated on 27 June 2011 containing 34,899 forward and 34,899 reverse sequences. A mass accuracy of ±5 ppm was used for precursor ions and 1 Da for product ions. Enzyme specificity was limited to trypsin or LysC/trypsin, with at least one LysC or tryptic (K- or R-containing) terminus required per peptide and up to four mis-cleavages allowed. Cysteine carboxamidomethylation was specified as a static modification, oxidation of methionine residues was allowed, and phosphorylation was allowed on serine, threonine, and tyrosine residues. Reverse decoy databases were included for all searches to estimate false discovery rates, and filtered using a 1% FDR in the Linear Discriminant module of Core. Peptides were also manually filtered using reagent-specific criteria. For each antibody reagent results were filtered to include only phosphopeptides matching the sequence motif(s) targeted by the antibodies included, as shown in Figure 1B. For total proteome analysis, peptides were further filtered to an overall 5% protein false discovery rate using the ProteinSieve module in Core. Phosphorylation site localization probability scores were determined using the AScore module of Core [68] and are included in Tables S2–S7.

All quantitative results were generated using Progenesis V4.1 (Waters Corporation) to extract the integrated peak area of the corresponding peptide assignments according to previously published protocols [52,55,56,69]. The Progenesis software incorporates a chromatographic alignment (or time warping) algorithm that performs multiple binary comparisons to generate an overall clustering strategy for the complete data set of all identified peptides on the basis of a mass precision. Extracted ion chromatograms for peptide ions that changed in abundance between samples were manually reviewed to ensure accurate quantitation either in Progenesis or using XCalibur software (version 2.0.7 SP1, Thermo Scientific). Peak areas were normalized using a log2 median normalization strategy in Progenesis as previously described [38,45,52,55,69]. For total proteome analysis, the sum intensity for all peptide ions identified for a particular protein was found and used to generate fold-change values. 

### 2.7. Data Analysis

Area proportional Venn diagrams were created using the Venn diagram generator at the Whitehead Institute for Biomedical Research Bioinformatics and Research Computing website. Datasets for each enrichment were compiled from six LC-MS/MS runs, duplicate analyses of the three samples, DMSO, SU11274, and Staurosporine. Percent overlaps between any two datasets A and B were calculated using the formula (% overlap AB = 100% − (% unique to A + % unique to B). Quantitative data was evaluated and clustered in Spotfire DecisionSite (TIBCO Software AB, Waltham, MA, USA, 2015, version 9.1.2). Protein interaction networks were derived from the Ingenuity Pathway Analysis (IPA) software package (Qiagen, Valencia, CA, USA, 2015). Core analyses were run on the entire phospho dataset as well as on subsets of the phospho data that showed changes in abundance with SU11274 or staurosporine treatment. Only direct interactions were used, with experimental and high confidence predicted interactions allowed. Protein nodes were color-coded by the fold-changes for all the peptides identified from that protein to indicate peptides that increased (green), decreased (red), did not change (grey) or both increased/decreased on the same protein (yellow) with the indicated treatment.

### 2.8. Western Blotting

Protein concentrations for lysate supernatants were determined by Bradford assay using Coomassie Plus Protein Assay Reagent (Life Technologies, Carlsbad, CA, #23236), and protein amounts were normalized between samples. Samples were mixed with SDS-PAGE sample buffer (Cell Signaling Technology, #7723) and run on 4%–20% gradient tris-glycine gels (Life Technologies). Proteins were transferred to nitrocellulose (Millipore, Billerica, MA) and blocked for 1 h in 5% nonfat dry milk (Sigma) in TBS. Primary antibodies were incubated in 5% BSA in TBS plus 0.1% Tween-20 (TBS-T) overnight at 4°C. Membranes were washed 3 times with TBS-T, incubated with anti-rabbit or anti-mouse secondary antibody (#5366 and #5470, Cell Signaling Technology) for 1 h at room temperature in 5% milk TBS-T, washed 3 times with TBS-T, dried, and developed on the Odyssey near-infrared imaging system (LI-COR, Lincoln, NE, USA). All antibodies used were from Cell Signaling Technology. For gel stains, gels were washed 3 times with deionized H_2_O, stained with GelCode Blue Stain Reagent (Thermo Scientific, #24592), and destained with deionized H_2_O.

## 3. Results

The experimental design is outlined in Figure 1A. MKN-45 gastric carcinoma cells were treated with DMSO, the c-Met inhibitor SU11274, or the PKC inhibitor staurosporine. Protein amounts for each sample were equalized and a small portion of each sample was used for total protein analysis. Phosphopeptides were enriched from samples using IMAC or Cell Signaling Technology Motif Antibodies. Antibodies used for each enrichment are indicated in Figure 1B and include phosphotyrosine (pY-1000), a mix of motif antibodies targeting phosphoserine and phosphothreonine peptides containing basic residues (Basophilic), a mix targeting phosphoserine and phosphothreonine peptides containing proline (Proline-Directed) and a mix of all available motif antibodies targeting phosphoserine and phosphothreonine (Ser/Thr Mix). More information for each reagent and sample data can be found on the Cell Signaling Technology website (Danvers, MA)

**Figure 2:**
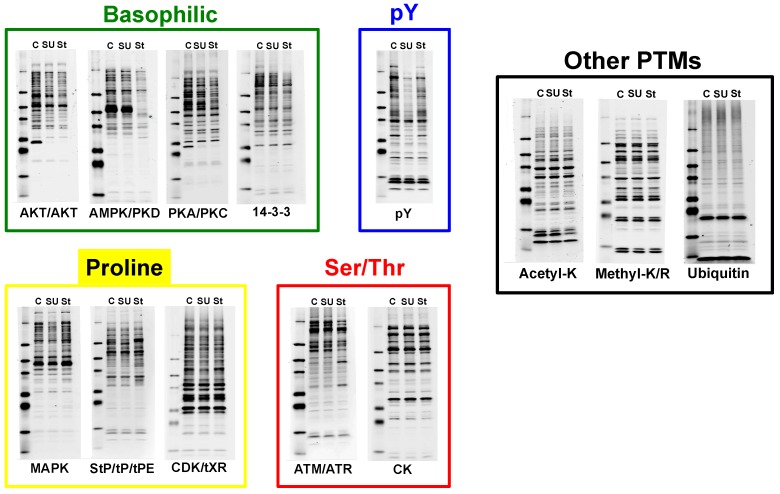
Western blot prescreen. Western blot prescreen of the MKN-45 samples with selected motif antibodies from Figure 1B. C = DMSO control, SU = SU11274, St = Staurosporine. Blots are organized by motif mix in which they were included.

The motif antibodies were used in Western blotting to assess changes in phosphorylation of proteins between control and treated samples (Figure 2). Individual motif antibodies are segregated by reagent in which they were used as indicated in Figure 1B. The Basophilic motif mix antibodies generally show decreased signal in response to staurosporine treatment relative to DMSO control. Phosphotyrosine pY-1000 shows a large decrease in signal with SU11274 treatment and a more modest decrease with staurosporine. Among the proline-directed motif antibodies, the response is more variable, with some decreases observed with SU11274 treatment and both decreases and increases with staurosporine. The changes for two antibodies included in the Ser/Thr mix were also variable, with a decrease in signal with staurosporine treatment using the ATM/ATR Substrate motif antibody, and little change with the CK2 Substrate motif antibody. In addition to the phosphorylation motif antibodies, samples were also screened using motif antibodies to other PTMs (Acetyl-Lysine, Mono-Methyl Arginine/Lysine, and Ubiquitin, black box). There were minor changes in levels of acetylated proteins with staurosporine treatment, and little or no change with ubiquitin or mono-methylation antibodies. Total protein levels were normalized for each sample as evidenced by gel stain as well as β-actin and Rab11 total protein control blots (Figure S2). Together, these data show the treatments performed were effective in changing phosphorylation patterns in MKN-45 cells.

Table S1 summarizes the LC-MS/MS data collected for both phosphopeptide enrichment and total protein analysis. In all cases, search results were filtered for a 1% false discovery rate. The enrichment specificity (% phosphopeptides/total peptides) is given for each sample, and ranged from 18% to 46% for antibody-enriched samples and over 95% for IMAC-enriched samples. The number of unique phosphopeptide identifications varied by antibody used and ranged from roughly 1500 (Proline-Directed) to over 3500 (Phosphotyrosine). IMAC enrichment yielded over 6900 unique phosphopeptides. The total proteome analysis yielded over 18,000 unique peptides per LC-MS/MS run after protein-level FDR filtering, corresponding to 4790 unique protein identifications. Full data tables for each enrichment and total proteome analysis are included as Tables S2–S7.

**Figure 3:**
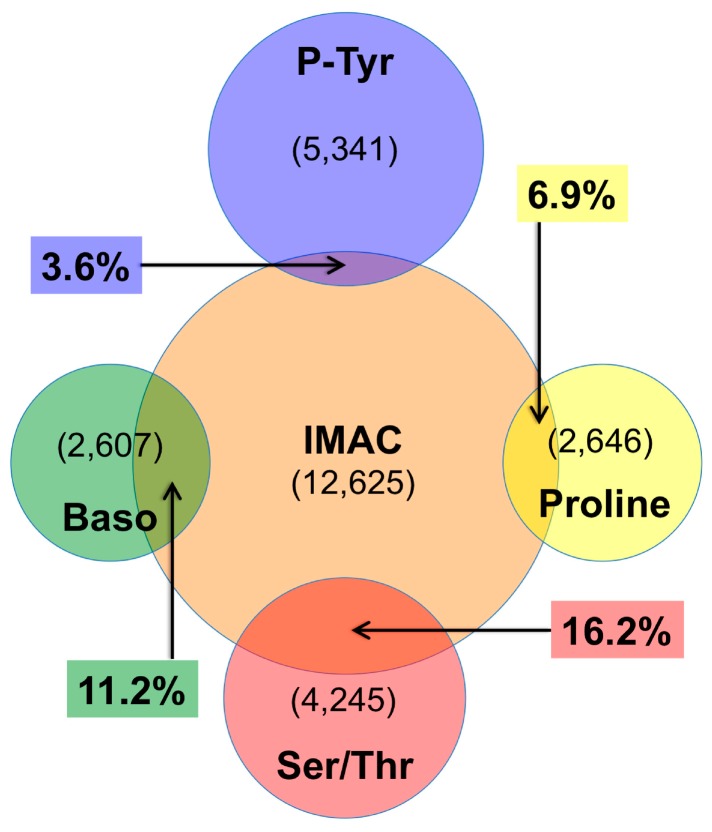
Venn diagram overlap of IMAC and antibody enrichments. The various enrichments are color-coded as in Figure 1. Venn diagrams are area proportional representing the number of unique proteins/sites identified in each enrichment (shown in parentheses). Arrows indicate the overlap for each antibody enrichment with the IMAC enrichment. Overlap between any two datasets A and B is calculated as 100% − (% unique to A + % unique to B).

An area proportional Venn diagram of the overlap between each of the antibody enrichments and the IMAC enrichment is shown in Figure 3. Comparisons were made using unique protein/site lists, though the overlaps observed were nearly identical when using the peptide sequences (data not shown). The number of unique protein-sites for each enrichment is shown in parentheses, and arrows indicate the overlap between the antibody enrichments and IMAC. For the Ser/Thr phosphopeptide enrichments, the overlaps with the IMAC dataset ranged from 6.9% to 16.2%. The overlap between phosphotyrosine and IMAC was lower, with only 3.6% of protein/sites in common between the two datasets (Figure 3).

Label-free quantitation was performed using Progenesis V4.1 for each dataset to find integrated peak areas of each peptide across all treatments. Peak areas were used to determine fold changes for each peptide in the SU11274 and staurosporine treated cells compared to the DMSO control. In cases where a change in relative abundance was observed for one or both treatments, the quantitative data was manually reviewed to ensure accuracy either in Progenesis or directly in the LC-MS/MS chromatogram files.

**Figure 4:**
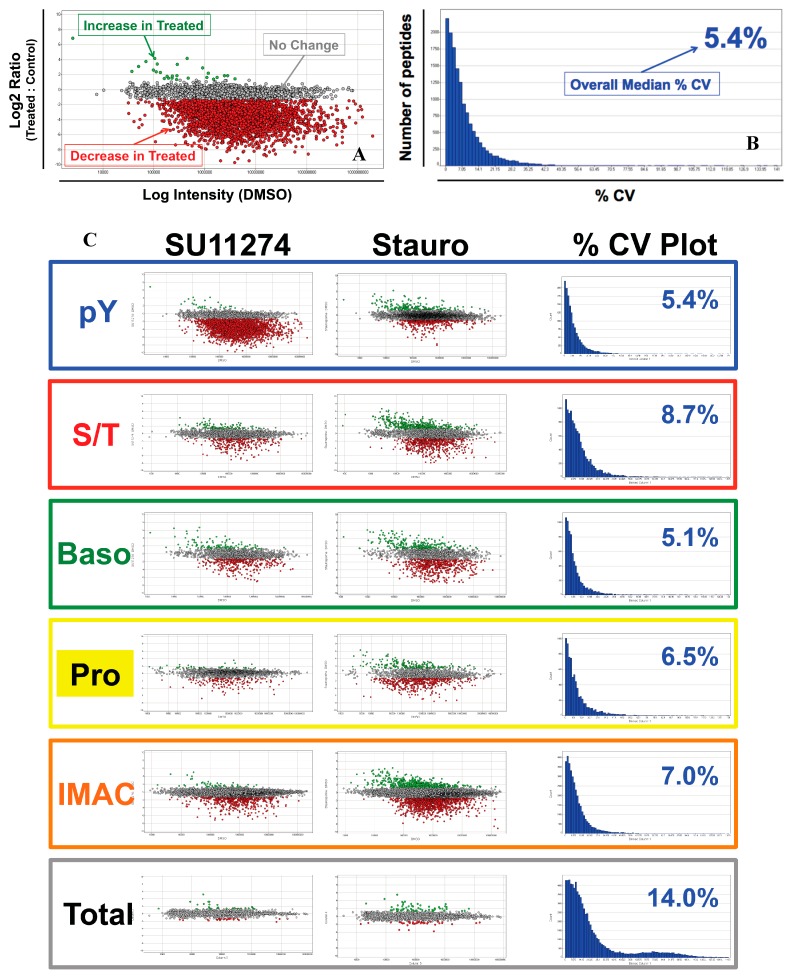

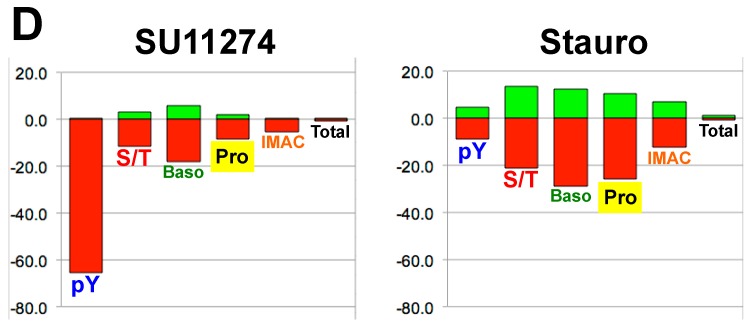
Quantitative analysis of proteomic data. (**A**) Representative Log2 ratio plot for proteomic data. Log2 ratio (Treated:Control) *versus* average intensity in DMSO control sample is plotted. Each point represents a unique peptide. Points are color-coded by fold change relative to control with >2.5-fold increase (green) > 2.5-fold decrease (red), and no change > 2.5-fold (grey); (**B**) Representative % CV histogram for proteomic data. % CV = ratio of standard deviation of the replicate measurements to the mean. All % CV values for a single enrichment were combined and plotted with number of peptides on the Y-axis and % CV on the X-axis. The overall median % CV for the dataset is shown in blue text; (**C**) Log2 ratio and % CV histogram plots are shown for each proteomic dataset as detailed in parts A and B, color-coded as in Figure 1 and Figure 2; (**D**) The percentages of unique peptides from each dataset that changed > 2.5-fold are plotted for SU11274 (left) and staurosporine (right) treatments. The enrichments are indicated below the bars.

Quantitative data was plotted using Spotfire DecisionSite (TIBCO Software AB) for each comparison. Example plots are shown in detail in Figure 4A,B. Each ratio plot (Figure 4A) gives the Log2 ratio (treated:control) on the Y-axis with the Log intensity in the DMSO control sample on the X-axis. Plots are color-coded to denote peptides that increased >2.5-fold with SU11274 or staurosporine treatment (**green**), decreased >2.5-fold with treatment (**red**), or did not change more than 2.5-fold (**grey**). Each CV Plot (Figure 4B) is a histogram with number of peptides on the Y-axis and % CV bins on the X-axis. Each plot was generated from CV measurements across all samples for a given enrichment. The median % CV is indicated for each plot. CV data for each individual sample is given in Tables S2–S7. Plots for each enrichment are shown in Figure 4C along with a histogram in the right hand column of Figure 4C plotting the number of measurements *versus* % CV for analytical replicate injections. For each enrichment, the overall median % CV is indicated in blue text. The median % CV values for the antibody and IMAC enrichments were comparable, ranging from 5.1% to 8.7%. The median % CV for the total protein analysis was slightly higher at 14.0%. For all antibody enrichments, the median % CV data suggest excellent analytical reproducibility in agreement with previous results (38).

Figure 4D shows the percentage of peptides for each comparison that increased or decreased relative to the DMSO control. For all phosphopeptide enrichments, there were more peptides that decreased with SU11274 treatment than increased. Of all comparisons, the phosphotyrosine enrichment showed the largest number of changes with SU11274 treatment, with 3400 peptides decreasing relative to control (65% of peptides). This is in agreement with the pY-1000 Western blot in Figure 2 that showed a profound decrease in signal with SU11274 treatment. The response to staurosporine, like the Western blots in Figure 2, was more varied, with 100s of peptides that both increased and decreased in abundance relative to control. Strikingly, there were very few changes to total protein levels with either treatment, demonstrating that at the 2-h time point most regulation occurs at the level of post-translational modification.

**Figure 5:**
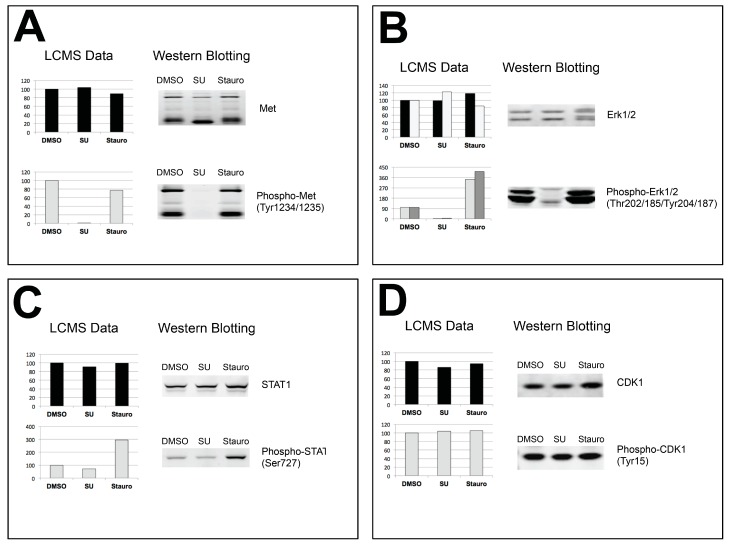
Western blot validation of proteomic data. Each panel shows relative intensity data from LC-MS/MS analysis on the left and Western blot validation on the right for c-Met (**A**), Erk1/Erk2 (**B**), Stat1 (**C**), and Cdk1 (**D**). Total proteome data and total protein blots are shown at the top of each panel (black bars in relative intensity plots), while phosphorylation site-specific proteomic data and blots are shown below (grey bars in relative intensity blots).

Selected proteins/sites were chosen for follow-up validation by Western blotting. Figure 5 shows both quantitative data from LC-MS/MS runs (left side) and Western blot results (right side) for c-Met (Figure 5A), Erk1/2 (Figure 5B), Stat1 (Figure 5C), and Cdk1 (Figure 5D). Additional validation blots and corresponding LC-MS/MS quantitative data are provided in Supplemental Figure S2. In all cases, there was excellent agreement between the quantitative data and the follow-up Western blots. For c-Met, the receptor tyrosine kinase responsible for MKN-45 cell growth and survival, there was no change in total protein levels as assessed by LC-MS/MS quantitation or Western blot. Phosphorylation of c-Met at Tyr1234/Tyr1235, however, was completely abrogated by SU11274 treatment, with little or no change in response to staurosporine (Figure 5A). Similarly, Erk1 and Erk2 total levels were relatively unaffected by treatment, while phosphorylation at Thr185/202 and Tyr 187/204 was down-regulated in response to SU11274 treatment. This phosphorylation actually increased with staurosporine treatment relative to control (Figure 5B). Total Stat1 levels did not change with either treatment relative to control, while Stat1 phosphorylation at Ser727 increased with staurosporine (Figure 5C). Cdk1 is shown as a control, as neither total levels nor phosphorylation at Tyr15 changed with either treatment (Figure 5D).

**Figure 6:**
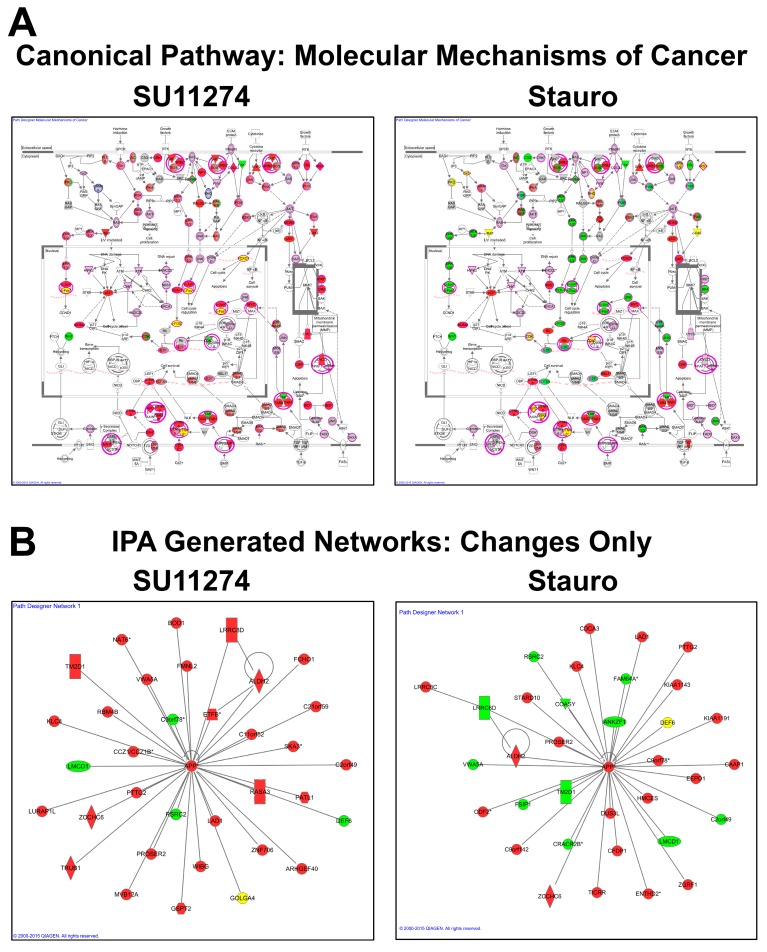
IPA protein interaction network analysis. The phosphoproteomic data from IMAC and antibody enrichments was combined and analyzed by Ingenuity Pathway Analysis (IPA) software. (**A**) Phosphoproteomic data mapped onto the IPA canonical network Molecular Mechanisms of Cancer for SU11274 (left) and staurosporine (right) treatments. Protein nodes highlighted in violet and/or grey were identified in the study. Proteins are color-coded with peptides that changed >2.5-fold up (green), down >2.5-fold (red), or both up and down >2.5-fold (yellow) with the indicated treatment. (**B**) IPA analysis was also run for each treatment including only proteins with peptides that changed >2.5-fold. Examples of networks generated *de novo* by IPA for SU11274 (left) and staurosporine (right) treatments are shown and color-coded as in A.

Examples of canonical pathways (Molecular Mechanisms of Cancer, Figure 6A) and *de novo*-generated IPA networks (Figure 6B) from the network analysis using Ingenuity Pathway Analysis software (IPA, Qiagen) are also provided to highlight possibilities for data analysis post-data acquisition. Pathway proteins are color-coded by fold change from the combined phosphorylation data (Tables S2–S6), with green nodes indicating proteins with peptides that increased with SU11274 or staurosporine treatment, red nodes indicating decreases, yellow nodes indicating proteins with peptides that both increase and decrease with treatment, and grey nodes indicating proteins with identified peptides that did not change with treatment. The Molecular Mechanisms of Cancer pathways in Figure 6A show deep coverage of proteins identified from the combined phosphoproteomic data. SU11274 treatment caused a decrease in peptide abundance for many proteins in the pathway, while there were both increases and decreases with staurosporine treatment. To generate the *de novo* networks in Figure 6B, only proteins with peptides that changed with treatment were used. Therefore, the composition of the two pathways differs between SU11274 and staurosporine treatment. Further examples of both IPA canonical pathways and IPA-generated networks are included as Supplemental IPA Data. 

**Figure 7:**
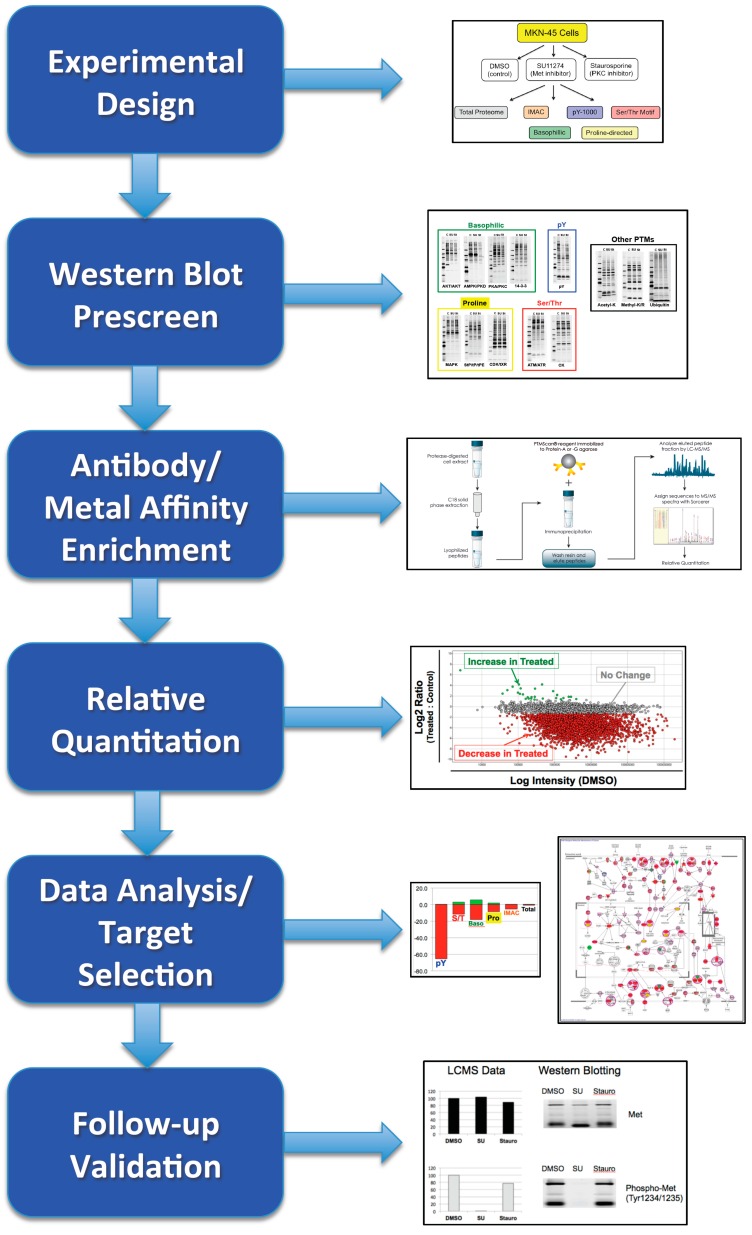
Start-to-finish proteomic analysis workflow. The steps taken for experimental design, prescreening, proteomic analysis, data analysis, and follow-up validation are shown with example images from each step. Together, these steps comprise an optimized workflow to maximize chances of a successful study to identify therapeutic targets, biomarkers, or critical signaling molecules.

A generalized experimental workflow for both PTM and total protein analysis is presented in Figure 7. Once an experimental system is chosen, cell lines, tissues, xenografts, or other biological material can be used as input. Preliminary Western blotting can be performed to ensure efficacy of treatment or difference between samples, and to help select the appropriate enrichment(s) to use for LC-MS/MS analysis. Enrichments can be performed in parallel, with greatest coverage of phosphorylation coming from use of both metal-affinity and antibody-based enrichments. If preliminary Western blots show changes with multiple PTM motif antibodies, those antibodies can be combined to allow for identification of greater numbers of peptides from a single LC-MS/MS run. If multiple different PTMs are to be investigated, the same sample can be sequentially immunoprecipitated with the antibodies of interest. Sequential enrichment provides the advantage of performing multiple analyses without the need to prepare fresh material for each. Relative quantitation can be performed using peak detection software such as Progenesis and fold changes determined. These quantitative results can then be pared down to select the most biologically relevant candidates for follow-up analysis. In cases where the best potential candidates are unclear, protein interaction network analysis can be used to help visualize relevant pathways and signaling areas affected by treatment.

## 4. Discussion

### 4.1. Experimental Design/Western Blot Prescreen

The MKN-45 gastric carcinoma cell line has been previously characterized as dependent on the receptor tyrosine kinase c-Met for growth and survival [39,62,64], and gastric carcinoma cells are also known to have dysregulation of the Ser/Thr protein kinase PKC [63]. As such, MKN-45 cells treated with inhibitors of c-Met (SU11274) and PKC (staurosporine) provide an ideal experimental system to investigate changes in phosphorylation using antibody-based and metal affinity enrichments. Western prescreening of protein lysates showed robust changes in tyrosine phosphorylation in response to the RTK inhibitor SU11274, as would be expected from treatment of an RTK-driven cell line. Multiple Western blots using antibodies against Ser/Thr phosphorylated motifs also showed significant changes in response to PKC inhibition with staurosporine (Figure 2). Together, these data suggested that the control and treated cells would be appropriate inputs for proteomic analysis of both Ser/Thr and Tyr phosphorylation. The widespread changes observed with multiple antibodies also demonstrated that combination of antibodies would be appropriate to maximize the number of phosphopeptide identifications observed for each sample. The observation of few changes with the other PTM antibodies (acetylation, methylation, ubiquitination) suggested proteomic profiling should focus on phosphorylation rather than these other PTMs. For other experimental systems, changes may be more profound with these PTM antibodies and would then be good candidates for proteomic analysis. The use of Western blot prescreening in this manner allows for design of a more focused, efficient proteomic study of changes that may occur between different cell lines or cells minus/plus treatment. 

### 4.2. IMAC versus Antibody Enrichment 

Sufficient sample was prepared for each treatment condition to allow antibody enrichment, IMAC enrichment, and total proteome analysis as summarized in Table S1. Both antibody and IMAC enrichments resulted in identification of thousands of phosphopeptides, many of which showed a response to treatment. Interestingly, the profile of phosphopeptides identified by the two enrichment methods was largely complimentary, with different pools of peptides identified by IMAC and antibody enrichment. As shown in Figure 3, the overlap between a given antibody enrichment and the IMAC enrichment ranged from roughly 16% with the all Ser/Thr antibody mix, to a low of only 3.6% with phosphotyrosine pY-1000. No fractionation of samples was performed prior to LC-MS/MS analysis in this study, and it is possible fractionation could increase the overlap between antibody and IMAC enrichment, however, the complementarity observed here between antibody enrichment and IMAC is consistent with other studies that fractionated samples prior to enrichment to compare the two methods [53,57,58,70,71]. These data demonstrate that to maximize the amount of information gleaned from each sample, both IMAC and antibody enrichment should be performed. This is particularly important for phosphotyrosine peptides, where the depth of coverage provided by antibody enrichment could not be recapitulated using IMAC alone. Parallel analysis of phosphoproteomic data using both methods would therefore give the greatest opportunity for discovery of critical phosphorylation events that could be used as therapeutic targets, biomarkers, or insights into the signaling basis of growth, development, and disease.

### 4.3. Data and Network Analysis 

In any proteomic study, the accuracy of quantitative data is critical to confidence in the changes observed. Past studies have proven that when performed properly, antibody enrichment prior to LC-MS/MS analysis combined with label-free quantitation can provide accurate, reproducible data across both analytical and biological replicates [38,55]. This point has again been demonstrated here, with excellent analytical reproducibility of the quantitation across the study, as evidenced by the % CV histograms (Figure 4C). Together, these data validate a label-free quantitative approach as a robust method for relative quantitation. It should be noted, however, that the same workflow described here could also be performed using isotopic labeling strategies such as SILAC or reductive dimethylation, or isobaric tagging methods such as iTRAQ or TMT [72,73,74,75,76].

The quantitative analysis of the phosphoproteomic data yielded hundreds of phosphopeptides that changed in abundance between treatment conditions. In agreement with the Western blotting data in Figure 2, the phosphotyrosine data showed decreases in relative abundance of many phosphopeptides in response to the c-Met inhibitor SU11274. In fact, across all phosphopeptide enrichments, the trend in the quantitative data is an overall decrease in phosphopeptide abundance with SU11274 treatment (Figure 4D). This quantitative data is in agreement with the known biology of the system, in which treatment of the c-Met-driven MKN-45 cells with the Met inhibitor SU11274 causes growth arrest and eventual cell death [39,41,55,59,62]. This growth arrest/cell death phenotype is mediated by inhibition of multiple signaling pathways, evidenced here by decreased phosphorylation of both tyrosine and serine/threonine sites in diverse signaling areas. Figure 6A shows some examples of inhibited signaling cascades related to cancer cell growth and survival, including signaling through Ras-Raf-Mek-Erk pathways, PI3K signaling, β-catenin signaling, and apoptosis pathways. Similar negative effects on signaling can also be visualized using IPA software for adherens junction signaling, integrin signaling, HGF signaling, and Paxillin signaling, among others (Supplemental IPA Data).

Interestingly, there was a more varied response to staurosporine, with peptides that both increased and decreased in abundance with treatment, as seen in Figure 4D. These increases and decreases in phosphopeptide levels with staurosporine treatment were again consistent with the Western blotting data in Figure 2. The selected basophilic motif antibodies show a decrease in phosphoprotein band intensity in response to staurosporine, while proline-directed and Ser/Thr motif antibodies showed phosphoproteins that both increased and decreased. These bidirectional changes were also seen in the network analysis as in Figure 6A,B where, in contrast to the SU11274 analysis, many proteins were observed with peptides that increased and decreased with staurosporine. Additional examples of the varied response to staurosporine can also be seen in the IPA networks shown in the Supplemental IPA Data.

In contrast to the thousands of phosphopeptide changes observed with SU11274 and staurosporine treatment, there was a relative paucity of changes at the total protein level. This can clearly be seen in the scatter plots in Figure 4C and the bar charts in Figure 4D. This is an important point, as many proteomic analyses currently focus on protein-level changes rather than changes to post-translational modifications. For many experimental systems, these protein-level analyses have yielded important insights into the biology of the system being studied. However, for these treatments at these time points (2 h post-treatment), focusing solely on total protein changes would have yielded very few if any actionable discoveries. This points to the necessity to determine the best approach prior to embarking on a large-scale LC-MS/MS-based study using tools such as the Western blotting prescreen in Figure 2.

### 4.4. Validation of Results

An important metric of the success of any study, proteomic or otherwise, is the ability to confirm the accuracy of results via independent testing. Here, we have assessed the accuracy of the LC-MS/MS data by comparing the results to follow-up Western blotting analysis (Figure 5 and Figure S2). Figure 5 shows four examples of follow-up data, comparing total protein and phosphopeptide signal intensities from the proteomic analysis to the western results using total protein and phosphorylation site-specific antibodies. In all cases, there was excellent agreement between the proteomic data and the Western blots. For the Met receptor, proteomic analysis showed no change in total c-Met protein levels with either treatment, but a large decrease in phosphorylation of c-Met at the activation loop sites Tyr1234/Tyr1235 [77,78] with SU11274. This result was recapitulated with the western data, with no change for the total protein blot, but an almost complete abrogation of phosphorylation at Tyr1234/Tyr1235 with SU11274 treatment. This is consistent with previous data [39,41] showing inhibition of c-Met phosphorylation in MKN-45 cells with SU11274.

The behavior of the MAP kinases Erk1 and Erk2 was also consistent between the proteomic and Western blotting data (Figure 5B). Erk total levels did not change to a great degree with either treatment. Phosphorylation of Erk1/Erk2 at the activation loop sites Thr202/Tyr204 (Erk1) or Thr185/Tyr187 (Erk2) decreased with SU11274 treatment in both phosphopeptide abundance and western data, consistent with previous results [39,41]. There was actually an increase in Erk phosphorylation with staurosporine treatment, again consistent in both methods, a phenomenon that has been reported for other cell types exposed to staurosporine [79,80,81,82]. Stat1 (Figure 5C) showed a similar pattern to Erk1/2 in response to staurosporine treatment, and no change with SU11274. Stat1 total levels were consistent throughout, while there was a robust increase in Stat1 Ser727 phosphorylation with staurosporine by both LC-MS/MS and Western blot. Initially this may seem surprising, as Stat1 Ser727 is a known substrate of the staurosporine target PKC [83], however, Ser727 can also be phosphorylated by Erk, which was activated by staurosporine in this system [84]. Cdk1 (Figure 5D) was also included in the validation as an example of a protein and a site (Tyr15) that did not change with either treatment, and once again, the proteomic data was fully consistent with the Western blotting analysis. Together, these data provide examples of the accuracy of the proteomic data as independently verified by Western blotting, consistent with previous studies that validated candidates identified through antibody-based enrichment and proteomic analysis [39,41,43,52,56,69].

## 5. Summary 

Figure 7 provides a template for organization of proteomic experiments to investigate both total protein levels as well as post-translational modifications. The first critical step is designing an experiment that will maximize the chance of finding targets for follow-up analysis. This includes selection of the proper cell line or tissue, time point for treatment, and dose of that treatment. These factors can be determined by preliminary experimentation when a well-defined control for efficacy exists, or through use of the Western blot prescreen with phosphorylation and other PTM-targeted motif antibodies. This prescreen also helps determine which PTM(s) and which antibodies would yield the most fruitful results in a proteomic experiment, by assessment of which antibodies show the greatest degree of change between samples. Once the experimental system has been optimized, the proteomic study can proceed, ideally incorporating both antibody-based and metal affinity enrichments to maximize coverage of the phosphoproteome. Relative quantitation between samples can be performed accurately irrespective of the enrichment used and also for total proteome analysis. The quantitative data will yield lists of potential targets for follow-up that can be narrowed by using fold change cutoffs, minimum signal intensity cutoffs, and variance (% CV) cutoffs. For experiments that show many changes between samples that could be difficult to interpret by review of the data tables alone, protein interaction network analysis can be performed to put the data within a known biological context. This data analysis will yield a list of candidates that can then be validated using independent biochemical follow-up experiments such as western blot, siRNA studies, overexpression of targets, or site-directed mutagenesis. Following this type of start-to-finish workflow maximizes the chance of finding critical regulators of the biological system under investigation as therapeutic targets, biomarkers, or insights into disease or normal cellular biology.

## Supplementary Materials

Supplementary materials can be accessed at: http://www.mdpi.com/
