# Supplementary material for: Complementary PTM Profiling of Drug Response in Human Gastric Carcinoma by Immunoaffinity and IMAC Methods with Total Proteome Analysis
Source: Proteomes. 2015 Aug 7;3(3):160–83. doi: 10.3390/proteomes3030160 (PMC5217380; doi:10.3390/proteomes3030160)
Supplement: Supplementary file 1 [file proteomes-03-00160-s001.zip › proteomes-03-00159-supplementary/proteomes-03-00159-supplementary.pdf]

Supplementary Materials

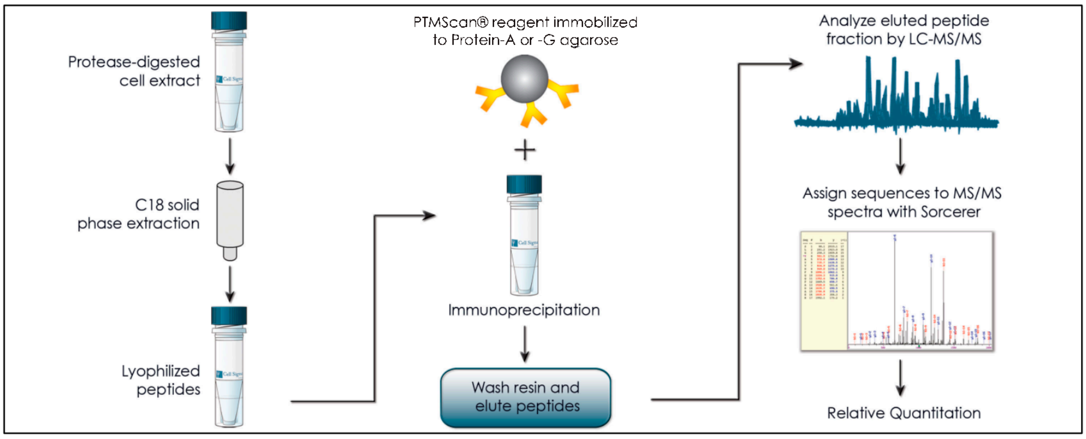

Figure S1. The PTMScan method [1]. Cell Lines, tissues, xenografts, or other biological materials are lysed, digested with Trypsin or LysC, and purified over C18 columns. Immunoaffinity Purification (IAP) is performed using Cell Signaling Technology Motif Antibodies. Eluted peptides are run in LC-MS/MS, and database searching/relative quantitation is performed.

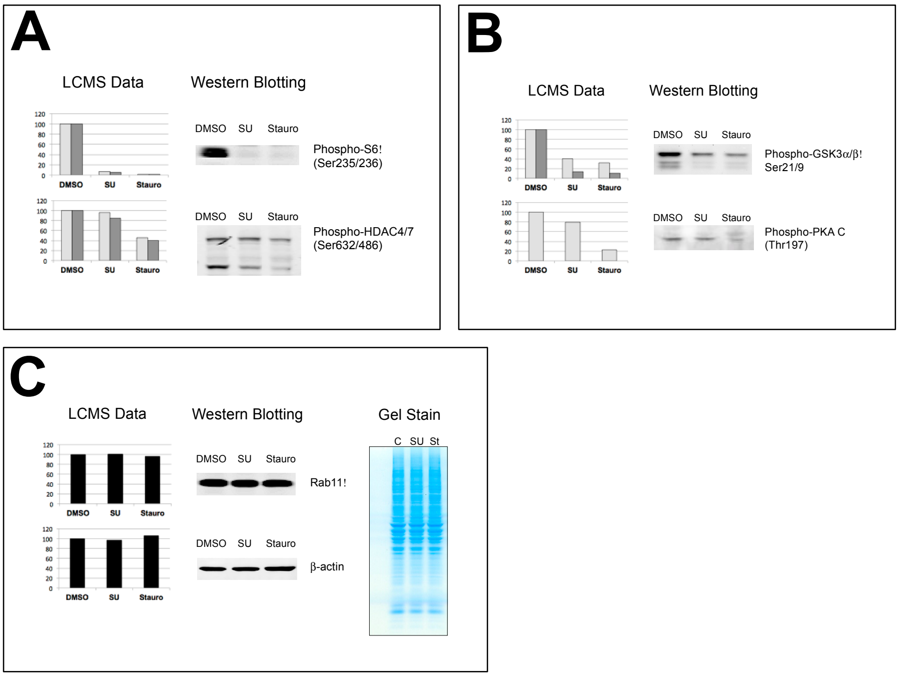

Figure S2. Validation western blotting. Samples used in proteomic analysis were run in western blot using the indicated antibodies. Each panel shows relative intensity data from LC-MS/MS analysis on the left and western blot validation on the right for Phospho-S6 (Ser235/Ser236) and HDAC 4/7 Ser632/Ser486 (panel **A**), Phospho GSK3 α/β (Ser21/9) and Phospho-PKA C Thr197 (panel **B**), Total Rab11 and β-actin (panel **C**). Panel **C** also includes a gel stain image from the western blot samples. Total proteome data is represented black bars in relative intensity plots, while phosphorylation site-specific proteomic data is shown with grey bars in relative intensity blots).

Table S1. LC-MS/MS information. Sample information, number of redundant and unique motif (phosphorylated) peptides and total peptides, false discovery rates and specificities are given for each LC-MS/MS run. Peptide and Protein level FDR is reported for the total proteome analysis. CHORUS ID number for each LC-MS/MS run is also included for access to raw data files.

Tables S2–S7. LC/MS-MS data tables. Each table includes a Column Definitions tab, a redundant list of all MS/MS identifications in the study with accompanying LC-MS/MS acquisition data and peptide assignment scoring data (Details Tab), a quantitative (Summary) tab non-redundant by protein/site, and a tab containing only peptides that changed in abundance with at least one treatment (Fold Change by Protein Type). Tables are filtered at a 1% FDR and for presence of the targeted phosphorylation motif where appropriate.

Reference

1. Rush, J.; Moritz, A.; Lee, K. A.; Guo, A.; Goss, V.L.; Spek, E.J.; Zhang, H.; Zha, X.M.; Polakiewicz, R.D.; Comb, M.J. Immunoaffinity profiling of tyrosine phosphorylation in cancer cells. *Nat. Biotechnol.* **2005**, *23*, 94–101.
