# Supplementary material for: Complementary PTM Profiling of Drug Response in Human Gastric Carcinoma by Immunoaffinity and IMAC Methods with Total Proteome Analysis
Source: Proteomes. 2015 Aug 7;3(3):160–83. doi: 10.3390/proteomes3030160 (PMC5217380; doi:10.3390/proteomes3030160)
Supplement: Supplementary file 1 [file proteomes-03-00160-s001.zip › proteomes-03-00159-supplementary/Supplemental_IPA_Data.zip!/Supplemental_IPA_Data/All_Proteins_Analysis/All_Proteins_Summary.pdf]

INGENUITY
PATHWAY ANALYSIS

Analysis Name: SU11274
Analysis Creation Date: 2015-03-10
Build version: 329271M
Content version: 21901358 (Release Date: 2014-12-09)

Analysis settings

View

Reference set: Ingenuity Knowledge Base (Genes + Endogenous Chemicals)

Relationship to include: Direct

Does not Include Endogenous Chemicals

Optional Analyses: My Pathways My List

Filter Summary:

Consider only relationships where

confidence = Experimentally Observed OR High (predicted)

Cutoff:

(c) 2000-2015 QIAGEN. All rights reserved.

Top Canonical Pathways

Name	p-value	Ratio
Germ Cell-Sertoli Cell Junction Signaling	1.26E-24	92/163 (0.564)
Epithelial Adherens Junction Signaling	4.18E-22	83/148 (0.561)
EIF2 Signaling	5.36E-22	97/188 (0.516)
Sertoli Cell-Sertoli Cell Junction Signaling	3.64E-21	95/186 (0.511)
Molecular Mechanisms of Cancer	1E-20	153/373 (0.41)

Top Upstream Regulators

Upstream Regulator	p-value of overlap	Predicted Activation State
TP53	9.45E-49	
HNF4A	1.03E-39	
MYC	1.80E-28	Inhibited
MYCN	2.07E-26	
E2F1	1.03E-23	

(c) 2000-2015 QIAGEN. All rights reserved.

INGENUITY® SYSTEMS

Top Diseases and Bio Functions

Diseases and Disorders

Name	p-value	# Molecules
Cancer	4.02E-157 - 2.85E-08	3850
Gastrointestinal Disease	1.42E-119 - 1.52E-09	2791
Hepatic System Disease	1.44E-75 - 4.06E-74	1768
Organismal Injury and Abnormalities	6.06E-55 - 2.24E-08	2231
Infectious Disease	6.51E-47 - 5.82E-14	733

Molecular and Cellular Functions

Name	p-value	# Molecules
Gene Expression	4.02E-77 - 4.21E-09	1118
Cellular Growth and Proliferation	4.05E-70 - 2.38E-08	1688
Cellular Assembly and Organization	6.71E-62 - 3.01E-08	1080
Cellular Function and Maintenance	6.71E-62 - 3.01E-08	1143
Cell Death and Survival	7.28E-52 - 2.66E-08	1543

Physiological System Development and Function

Name	p-value	# Molecules
Organismal Survival	7.03E-49 - 7.87E-09	1053
Connective Tissue Development and Function	1.10E-24 - 8.94E-09	473
Embryonic Development	2.70E-23 - 1.61E-08	723
Tissue Development	3.67E-21 - 1.61E-08	903
Nervous System Development and Function	5.63E-19 - 1.61E-08	551

(c) 2000-2015 QIAGEN. All rights reserved.

Top Tox Functions

Assays: Clinical Chemistry and Hematology

Name	p-value	# Molecules
Increased Levels of Albumin	4.03E-02 - 2.01E-01	3
Increased Levels of AST	1.07E-01 - 3.61E-01	10
Decreased Levels of Potassium	2.01E-01 - 5.92E-01	2
Increased Levels of Alkaline Phosphatase	2.51E-01 - 4.90E-01	25
Decreased Levels of Albumin	2.64E-01 - 3.61E-01	6

Cardiotoxicity

Name	p-value	# Molecules
Cardiac Necrosis/Cell Death	1.52E-05 - 5.92E-01	87
Congenital Heart Anomaly	6.50E-05 - 5.49E-01	70
Cardiac Hypertrophy	9.95E-05 - 5.02E-01	118
Cardiac Inflammation	9.39E-04 - 3.47E-01	30
Cardiac Hypoplasia	1.26E-02 - 2.01E-01	21

Hepatotoxicity

Name	p-value	# Molecules
Liver Hyperplasia/Hyperproliferation	1.44E-75 - 5.92E-01	1768
Hepatocellular Carcinoma	1.02E-06 - 2.01E-01	193
Liver Necrosis/Cell Death	1.87E-05 - 5.92E-01	101
Liver Proliferation	1.53E-04 - 4.90E-01	76
Liver Hypoplasia	5.87E-04 - 5.87E-04	19

Nephrotoxicity

(c) 2000-2015 QIAGEN. All rights reserved.

Name	p-value	# Molecules
Renal Necrosis/Cell Death	1.49E-10 - 1.00E00	166
Renal Proliferation	2.56E-07 - 3.01E-01	86
Renal Inflammation	8.74E-05 - 1.00E00	65
Renal Nephritis	8.74E-05 - 1.00E00	65
Renal Dysplasia	4.03E-02 - 3.26E-01	7

Top Regulator Effect Networks

ID	Regulators	Diseases & Functions	Consistency Score
1	GFI1	autophagy, cell survival, colony formation (+10 more)	16.667
2	IRF4	apoptosis of lung cancer cell lines (+11 more)	14.230
3	GMNN	apoptosis of fibroblast cell lines (+9 more)	7.071
4	NFYA	cell movement of breast cancer cell lines (+5 more)	6.667
5	SMARCB1	apoptosis of fibroblast cell lines (+7 more)	6.633

Top Networks

ID	Associated Network Functions	Score
1	Amino Acid Metabolism, Molecular Transport, Small Molecule Biochemistry	28
2	Cellular Assembly and Organization, Cell Cycle, DNA Replication, Recombination, and Repair	28
3	Infectious Disease, Protein Degradation, Protein Synthesis	28
4	Cancer, Cell Cycle, Cell Death and Survival	28
5	Cell Death and Survival, Cell-To-Cell Signaling and Interaction, Drug Metabolism	28

Top Tox Lists

(c) 2000-2015 QIAGEN. All rights reserved.

Name	p-value	Ratio
Renal Necrosis/Cell Death	1.85E-13	165/483 (0.342)
Mechanism of Gene Regulation by Peroxisome Proliferators via PPARα	2.52E-08	43/95 (0.453)
Liver Necrosis/Cell Death	5.75E-08	93/275 (0.338)
PPARα/RXRα Activation	1.48E-07	67/183 (0.366)
RAR Activation	1.55E-07	65/176 (0.369)

(c) 2000-2015 QIAGEN. All rights reserved.

INGENUITY® SYSTEMS

Top My Lists

Name	p-value	Ratio

Top My Pathways

Name	p-value	Ratio

Top Molecules

Other up-regulated

Molecules	Exp. Value	Exp. Chart
ABCA2	↑2.000	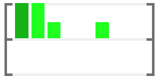
ADAM17	↑2.000	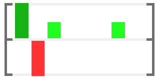
ADSL	↑2.000	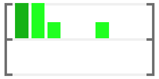
AFTPH	↑2.000	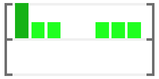
AKAP17A	↑2.000	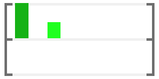
ALG3	↑2.000	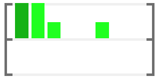
ANO6	↑2.000	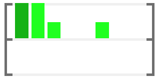
ARID3A	↑2.000	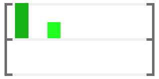

(c) 2000-2015 QIAGEN. All rights reserved.

ATL2	↑2.000	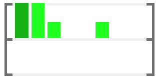
ATP6V1E1	↑2.000	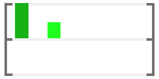

Other down-regulated

Molecules	Exp. Value	Exp. Chart
ZZEF1	↓-2.000	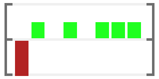
ZYX	↓-2.000	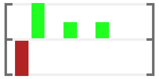
ZNRF2	↓-2.000	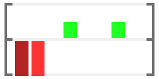
ZNF706	↓-2.000	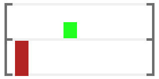
ZNF622	↓-2.000	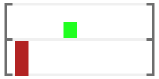
ZNF609	↓-2.000	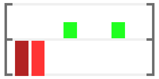
ZNF598*	↓-2.000	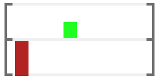
ZNF395	↓-2.000	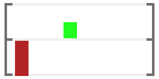
ZNF318	↓-2.000	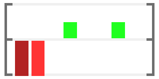
ZNF185*	↓-2.000	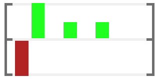

(c) 2000-2015 QIAGEN. All rights reserved.
