# Supplementary material for: Complementary PTM Profiling of Drug Response in Human Gastric Carcinoma by Immunoaffinity and IMAC Methods with Total Proteome Analysis
Source: Proteomes. 2015 Aug 7;3(3):160–83. doi: 10.3390/proteomes3030160 (PMC5217380; doi:10.3390/proteomes3030160)
Supplement: Supplementary file 1 [file proteomes-03-00160-s001.zip › proteomes-03-00159-supplementary/Supplemental_IPA_Data.zip!/Supplemental_IPA_Data/ChangesOnly_Stauro/ChangesOnly_Stauro_Summary.pdf]

INGENUITY PATHWAY ANALYSIS

Analysis Name: Stauro
Analysis Creation Date: 2015-03-11
Build version: 329271M
Content version: 21901358 (Release Date: 2014-12-09)

Analysis settings

View

Reference set: Ingenuity Knowledge Base (Genes + Endogenous Chemicals)

Relationship to include: Direct

Does not Include Endogenous Chemicals

Optional Analyses: My Pathways My List

Filter Summary:

Consider only relationships where

confidence = Experimentally Observed OR High (predicted)

Cutoff:

Other = 0.990

(c) 2000-2015 QIAGEN. All rights reserved.

Top Canonical Pathways

Name	p-value	Ratio
Integrin Signaling	4.77E-17	56/202 (0.277)
RhoA Signaling	1.54E-16	42/124 (0.339)
Signaling by Rho Family GTPases	3.17E-16	60/236 (0.254)
ERK/MAPK Signaling	1.43E-15	52/191 (0.272)
Sertoli Cell-Sertoli Cell Junction Signaling	1.95E-15	51/186 (0.274)

Top Upstream Regulators

Upstream Regulator	p-value of overlap	Predicted Activation State
TP53	1.16E-31	
MYCN	1.34E-16	
MYC	5.19E-15	Inhibited
HNF4A	2.70E-14	
NUPR1	4.81E-13	

(c) 2000-2015 QIAGEN. All rights reserved.

Top Diseases and Bio Functions

Diseases and Disorders

Name	p-value	# Molecules
Cancer	3.56E-84 - 1.24E-06	1578
Gastrointestinal Disease	2.62E-72 - 3.21E-07	1192
Hepatic System Disease	1.29E-51 - 2.77E-51	785
Organismal Injury and Abnormalities	7.39E-37 - 1.24E-06	939
Reproductive System Disease	4.06E-26 - 1.24E-06	786

Molecular and Cellular Functions

Name	p-value	# Molecules
Cellular Assembly and Organization	5.17E-47 - 1.20E-06	489
Cellular Function and Maintenance	5.17E-47 - 4.88E-07	580
Cellular Growth and Proliferation	3.02E-45 - 8.62E-07	745
Cell Cycle	2.83E-35 - 1.08E-06	384
Gene Expression	6.40E-34 - 7.78E-07	463

Physiological System Development and Function

Name	p-value	# Molecules
Organismal Survival	7.88E-26 - 7.88E-26	457
Tissue Development	1.98E-20 - 9.98E-07	497
Embryonic Development	4.28E-19 - 1.33E-06	347
Connective Tissue Development and Function	1.45E-18 - 7.09E-07	229
Tissue Morphology	1.51E-15 - 1.33E-06	349

(c) 2000-2015 QIAGEN. All rights reserved.

INGENUITY® SYSTEMS

Top Tox Functions

Assays: Clinical Chemistry and Hematology

Name	p-value	# Molecules
Increased Levels of Hematocrit	4.39E-02 - 4.39E-02	13
Decreased Levels of Potassium	7.97E-02 - 2.83E-01	2
Increased Levels of Albumin	7.97E-02 - 5.26E-01	3
Increased Levels of Alkaline Phosphatase	1.53E-01 - 5.37E-01	8
Increased Levels of Red Blood Cells	1.80E-01 - 1.80E-01	11

Cardiotoxicity

Name	p-value	# Molecules
Cardiac Necrosis/Cell Death	8.60E-07 - 2.85E-01	48
Cardiac Hypertrophy	1.12E-06 - 4.95E-01	59
Cardiac Proliferation	3.72E-05 - 3.08E-01	21
Cardiac Fibrosis	2.14E-03 - 5.33E-01	29
Cardiac Dilation	9.45E-03 - 2.20E-01	14

Hepatotoxicity

Name	p-value	# Molecules
Liver Hyperplasia/Hyperproliferation	1.29E-51 - 4.41E-01	785
Liver Necrosis/Cell Death	1.36E-06 - 3.97E-01	54
Hepatocellular Carcinoma	3.60E-06 - 2.20E-01	90
Liver Proliferation	1.41E-04 - 2.20E-01	35
Liver Hypoplasia	1.51E-04 - 1.51E-04	12

Nephrotoxicity

(c) 2000-2015 QIAGEN. All rights reserved.

Name	p-value	# Molecules
Renal Proliferation	6.76E-11 - 5.72E-01	51
Renal Necrosis/Cell Death	1.86E-05 - 1.00E00	68
Renal Inflammation	5.36E-03 - 5.00E-01	27
Renal Nephritis	5.36E-03 - 5.00E-01	27
Glomerular Injury	1.06E-02 - 3.92E-01	16

Top Regulator Effect Networks

ID	Regulators	Diseases & Functions	Consistency Score
1	ARNT, HSF1, MYOD1	incidence of liver tumor (+2 more)	3.175
2	E2F1, MYC, SP1	progression of carcinoma, translation of RNA	2.182
3	E2F1	osteosarcoma, progression of carcinoma	1.732
4	TFAP4	senescence of fibroblast cell lines	1.155
5	MKL1	apoptosis of neurons	0.500

Top Networks

ID	Associated Network Functions	Score
1	Cardiovascular Disease, Cell Death and Survival, Cellular Assembly and Organization	42
2	Auditory Disease, Developmental Disorder, Hereditary Disorder	42
3	Cancer, Gastrointestinal Disease, Cell Morphology	40
4	Protein Synthesis, Gene Expression, RNA Post-Transcriptional Modification	40
5	Cellular Assembly and Organization, Developmental Disorder, Hereditary Disorder	40

Top Tox Lists

(c) 2000-2015 QIAGEN. All rights reserved.

INGENUITY® SYSTEMS

Name	p-value	Ratio
Mechanism of Gene Regulation by Peroxisome Proliferators via PPARα	6.69E-08	25/95 (0.263)
Liver Necrosis/Cell Death	2E-07	48/275 (0.175)
Cardiac Necrosis/Cell Death	1.16E-06	45/266 (0.169)
Renal Necrosis/Cell Death	5.83E-06	67/483 (0.139)
Cardiac Hypertrophy	1.13E-05	56/389 (0.144)

(c) 2000-2015 QIAGEN. All rights reserved.

INGENUITY® SYSTEMS

Top My Lists

Name	p-value	Ratio

Top My Pathways

Name	p-value	Ratio

Top Molecules

Other up-regulated

Molecules	Exp. Value	Exp. Chart
3,4-dihydro-5[4-(1-piperindinyl)butoxy]-1(2H)-isoquinoline	↑2.000	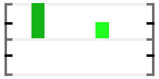
5-methoxypsoralen	↑2.000	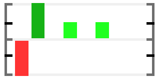
ABCA2	↑2.000	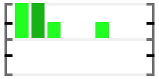
ADAR	↑2.000	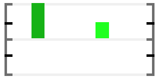
ADCY6	↑2.000	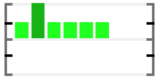
ADD3	↑2.000	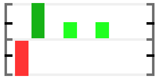
ADSL	↑2.000	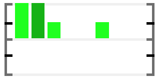
AFAP1L2	↑2.000	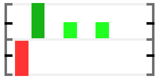

(c) 2000-2015 QIAGEN. All rights reserved.

INGENUITY® SYSTEMS

AFP	↑2.000	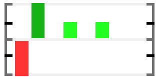
AIM1	↑2.000	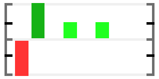

Other down-regulated

Molecules	Exp. Value	Exp. Chart
ZXDC	↓-2.000	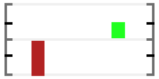
ZNRF2	↓-2.000	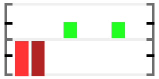
ZNF827	↓-2.000	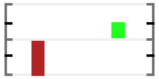
ZNF687	↓-2.000	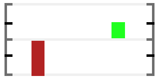
ZNF644	↓-2.000	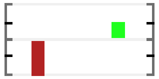
ZNF638*	↓-2.000	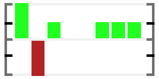
ZNF609	↓-2.000	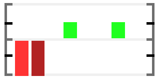
ZNF318	↓-2.000	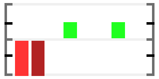
ZMYND8*	↓-2.000	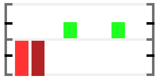
ZMYM3*	↓-2.000	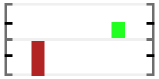

(c) 2000-2015 QIAGEN. All rights reserved.

INGENUITY® SYSTEMS
