# Supplementary material for: Complementary PTM Profiling of Drug Response in Human Gastric Carcinoma by Immunoaffinity and IMAC Methods with Total Proteome Analysis
Source: Proteomes. 2015 Aug 7;3(3):160–83. doi: 10.3390/proteomes3030160 (PMC5217380; doi:10.3390/proteomes3030160)
Supplement: Supplementary file 1 [file proteomes-03-00160-s001.zip › proteomes-03-00159-supplementary/Supplemental_IPA_Data.zip!/Supplemental_IPA_Data/ChangesOnly_SU11274/ChangesOnly_SU11274_Summary.pdf]

Analysis Name: SU11274
Analysis Creation Date: 2015-03-11
Build version: 329271M
Content version: 21901358 (Release Date: 2014-12-09)

Analysis settings

View

Reference set: Ingenuity Knowledge Base (Genes + Endogenous Chemicals)

Relationship to include: Direct

Does not Include Endogenous Chemicals

Optional Analyses: My Pathways My List

Filter Summary:

Consider only relationships where

confidence = Experimentally Observed OR High (predicted)

Cutoff:

Other = 0.990

(c) 2000-2015 QIAGEN. All rights reserved.

Top Canonical Pathways

Name	p-value	Ratio
Actin Cytoskeleton Signaling	1.57E-26	71/221 (0.321)
Sertoli Cell-Sertoli Cell Junction Signaling	4.19E-25	63/186 (0.339)
Germ Cell-Sertoli Cell Junction Signaling	1.82E-24	58/163 (0.356)
Integrin Signaling	1.93E-24	65/202 (0.322)
Epithelial Adherens Junction Signaling	5.88E-21	51/148 (0.345)

Top Upstream Regulators

Upstream Regulator	p-value of overlap	Predicted Activation State
TP53	7.77E-36	
FOS	6.53E-12	
HNF4A	1.77E-11	
MYCN	1.81E-11	
MYC	4.68E-11	Inhibited

(c) 2000-2015 QIAGEN. All rights reserved.

INGENUITY® SYSTEMS

Top Diseases and Bio Functions

Diseases and Disorders

Name	p-value	# Molecules
Cancer	1.23E-75 - 2.32E-07	1504
Gastrointestinal Disease	2.38E-56 - 1.66E-07	1111
Hepatic System Disease	4.63E-37 - 1.03E-36	715
Organismal Injury and Abnormalities	3.35E-36 - 2.26E-07	887
Infectious Disease	1.11E-28 - 8.39E-09	318

Molecular and Cellular Functions

Name	p-value	# Molecules
Cellular Assembly and Organization	1.28E-62 - 2.15E-07	511
Cellular Function and Maintenance	1.28E-62 - 2.15E-07	623
Cellular Growth and Proliferation	6.19E-47 - 2.23E-07	727
Cellular Movement	5.20E-44 - 1.33E-07	488
Cell Morphology	8.02E-39 - 1.07E-07	526

Physiological System Development and Function

Name	p-value	# Molecules
Organismal Survival	4.30E-26 - 4.88E-08	439
Nervous System Development and Function	3.32E-23 - 1.12E-07	333
Tissue Development	3.32E-23 - 1.49E-07	513
Connective Tissue Development and Function	5.09E-17 - 1.27E-07	218
Organismal Development	4.23E-16 - 2.18E-07	501

(c) 2000-2015 QIAGEN. All rights reserved.

INGENUITY SYSTEMS

Top Tox Functions

Assays: Clinical Chemistry and Hematology

Name	p-value	# Molecules
Increased Levels of Albumin	5.83E-03 - 6.44E-01	5
Increased Levels of Hematocrit	3.30E-02 - 3.30E-02	13
Increased Levels of ALT	1.06E-01 - 1.06E-01	5
Increased Levels of Creatinine	1.07E-01 - 3.20E-01	8
Increased Levels of AST	1.36E-01 - 4.71E-01	5

Cardiotoxicity

Name	p-value	# Molecules
Cardiac Inflammation	2.24E-06 - 5.81E-01	14
Cardiac Hypertrophy	5.75E-05 - 6.01E-01	55
Cardiac Necrosis/Cell Death	7.00E-05 - 4.87E-01	40
Cardiac Fibrosis	4.74E-03 - 5.09E-01	27
Cardiac Proliferation	5.44E-03 - 2.91E-01	16

Hepatotoxicity

Name	p-value	# Molecules
Liver Hyperplasia/Hyperproliferation	4.63E-37 - 5.79E-01	715
Liver Necrosis/Cell Death	1.94E-06 - 2.72E-01	53
Liver Hypoplasia	2.07E-05 - 2.07E-05	13
Liver Proliferation	1.67E-04 - 1.47E-01	37
Hepatocellular Carcinoma	1.71E-04 - 3.28E-01	81

Nephrotoxicity

(c) 2000-2015 QIAGEN. All rights reserved.

INGENUITY SYSTEMS

Name	p-value	# Molecules
Renal Necrosis/Cell Death	3.44E-09 - 5.09E-01	80
Renal Proliferation	1.04E-04 - 4.27E-01	39
Glomerular Injury	3.79E-04 - 6.44E-01	23
Renal Inflammation	8.87E-04 - 4.71E-01	33
Renal Nephritis	8.87E-04 - 4.71E-01	33

Top Regulator Effect Networks

ID	Regulators	Diseases & Functions	Consistency Score
1	DDX17	apoptosis of fibroblast cell lines (+12 more)	21.500
2	FOXC2	colony formation of cells, Growth Failure (+4 more)	7.348
3	GMNN	branching of cells, cell survival (+8 more)	7.000
4	HIPK2	development of neurons, formation of lamellipodia (+4 more)	5.669
5	HIPK2, NFYA, SATB1, SMARCB1	colon tumor, congenital heart disease (+5 more)	5.425

Top Networks

ID	Associated Network Functions	Score
1	Cell Death and Survival, Cellular Assembly and Organization, Developmental Disorder	43
2	Cellular Function and Maintenance, Skeletal and Muscular System Development and Function, Tissue Development	43
3	Cellular Assembly and Organization, Cellular Function and Maintenance, Lipid Metabolism	40
4	Cancer, Cell Cycle, Cellular Assembly and Organization	40
5	RNA Post-Transcriptional Modification, RNA Damage and Repair, Molecular Transport	40

Top Tox Lists

(c) 2000-2015 QIAGEN. All rights reserved.

INGENUITY SYSTEMS

Name	p-value	Ratio
Renal Necrosis/Cell Death	8.61E-11	79/483 (0.164)
Mechanism of Gene Regulation by Peroxisome Proliferators via PPARα	1.32E-09	27/95 (0.284)
PPARα/RXRα Activation	3.64E-09	39/183 (0.213)
Liver Necrosis/Cell Death	2.1E-08	49/275 (0.178)
TGF-β Signaling	3.11E-06	21/90 (0.233)

(c) 2000-2015 QIAGEN. All rights reserved.

Top My Lists

Name	p-value	Ratio

Top My Pathways

Name	p-value	Ratio

Top Molecules

Other up-regulated

Molecules	Exp. Value	Exp. Chart
ABCA2	↑2.000	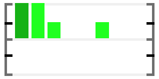
ADAM17	↑2.000	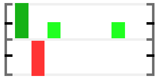
ADSL	↑2.000	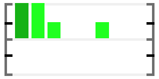
AFTPH	↑2.000	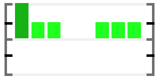
AKAP17A	↑2.000	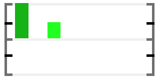
ALG3	↑2.000	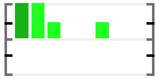
ANO6	↑2.000	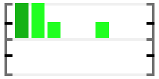
ARID3A	↑2.000	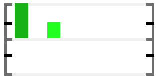

(c) 2000-2015 QIAGEN. All rights reserved.

INGENUITY® SYSTEMS

ATL2	↑2.000	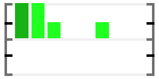
ATP6V1E1	↑2.000	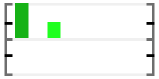

Other down-regulated

Molecules	Exp. Value	Exp. Chart
ZZEF1	↓-2.000	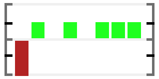
ZYX	↓-2.000	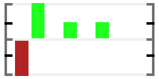
ZNRF2	↓-2.000	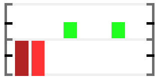
ZNF706	↓-2.000	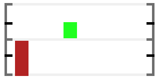
ZNF622	↓-2.000	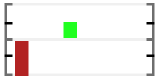
ZNF609	↓-2.000	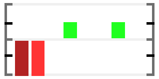
ZNF598*	↓-2.000	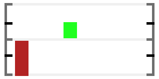
ZNF395	↓-2.000	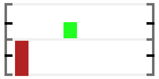
ZNF318	↓-2.000	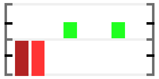
ZNF185*	↓-2.000	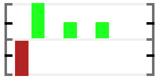

(c) 2000-2015 QIAGEN. All rights reserved.
